# MiR-146a G/C rs2910164 variation in South African Indian and Caucasian patients with psoriatic arthritis

**DOI:** 10.1186/s12881-018-0565-1

**Published:** 2018-03-27

**Authors:** Ajesh B. Maharaj, Pragalathan Naidoo, Terisha Ghazi, Naeem S. Abdul, Shanel Dhani, Taskeen F. Docrat, Prithiksha Ramkaran, Paul-Peter Tak, Niek de Vries, Anil A. Chuturgoon

**Affiliations:** 1 Department of Internal Medicine, Prince Mshiyeni Memorial Hospital and School of Clinical Medicine, College of Health Sciences, Nelson R Mandela School of Medicine, University of KwaZulu-Natal, Durban, South Africa; 2 Division of Clinical Immunology and Rheumatology, Academic Medical Center, University of Amsterdam, Amsterdam, Netherlands; 3 School of Laboratory Medicine and Medical Sciences, Discipline of Medical Biochemistry and Chemical Pathology, University of KwaZulu-Natal, George Campbell Building – South Entrance, 3rd Floor, King George V Avenue, Howard College Campus, Durban, 4001 South Africa

**Keywords:** miR-146a rs2910164, Psoriatic arthritis, South African Caucasian and Indian population

## Abstract

**Background:**

Psoriasis and psoriatic arthritis (PsA) are inflammatory associated autoimmune disorders. MicroRNA (miR)-146a plays a crucial role in regulating inflammation. A single nucleotide polymorphism in the miR-146a gene (rs2910164), aberrantly alters its gene expression and linked with the pathogenesis of several disorders, including psoriasis and PsA. In South Africa, psoriasis and PsA are extremely rare in the indigenous African population and most common in both the Indian and Caucasian population. The aim of this study was to investigate whether the miR-146a rs2910164 contributes towards psoriasis and PsA development in South African Indian and Caucasian patients.

**Methods:**

South African Indian (*n* = 84) and Caucasian (*n* = 32) PsA patients (total *n* = 116) and healthy control subjects (Indian: *n* = 62 and Caucasian: *n* = 38; total *n* = 100) were recruited in the study. DNA was extracted from whole blood taken from all subjects, and genotyped for the miR-146a rs2910164 using polymerase chain reaction-restriction fragment length polymorphism (PCR-RFLP). Data for laboratory parameters were obtained from pathology reports. The consulting rheumatologist collected all other clinical data.

**Results:**

***Unstratified data (Caucasians + Indians)*:** A significant decrease in C-reactive protein (CRP) levels in PsA patients was observed (CRP monitored at inclusion vs. after 6 months of treatment) (18.95 ± 2.81 mg/L vs. 9.68 ± 1.32 mg/L, *p* = 0.0011). The miR-146a rs2910164 variant C-allele frequency in PsA patients was significantly higher vs. healthy controls (35.78% vs. 26% respectively, *p* = 0.0295, OR = 1.59 95% CI 1.05–2.40). ***Stratified data (Indians)*:** The variant C-allele frequency in Indian PsA patients was significantly higher vs. healthy Indian controls (35.71% vs. 22.58%, *p* = 0.0200, OR = 1.91 95% CI 1.13–3.22). ***Stratified data (Caucasians)*:** The variant C-allele frequency distribution between Caucasian PsA patients and healthy Caucasian controls was similar.

**Conclusion:**

The rs2910164 variant C-allele may play a role in the progression of PsA in the South African Indian population. The main limitation in this study was the small sample size in the case-control cohorts, with a low overall statistical power (post-hoc power analysis = 19%).

## Background

Psoriasis is a chronic immune-mediated inflammatory skin disease triggered by a broad spectrum of genetic and environmental factors [1], and characterized by hyperproliferative keratinocytes, and aberrantly increased T lymphocyte (T-cell) activation and T-helper cell type 1 (T_H_1) cytokine production [2]. Psoriasis is associated with an inflammatory arthritis, namely psoriatic arthritis (PsA) [3]. Around 30% (6–42%) of patients with psoriasis develop PsA [4–6].

MicroRNAs (miRs) are small non-coding RNAs that control gene expression at the post-transcriptional level by negatively regulating the processing, stability, and translation of mRNA. The highly conserved “seed” region of miRs, composed of 2–7 nucleotides and located at the 5′-untranslated region (5’-UTR), binds to the 3’-UTR of their target mRNA to elicit their aforementioned functions [7]. MiRs play an invaluable role in regulating physiological processes in the body, including cell cycle progression, cell differentiation, metabolism and apoptosis [8]. When miRs are aberrantly expressed, due to single nucleotide polymorphisms (SNPs) within miR encoding genes or environmental factors (pollution, teratogens, and smoking), they can also contribute towards the pathogenesis of several inflammatory disorders [9].

MiR-146a is located on human chromosome 5q34 and plays an important role in regulating immune and inflammatory response pathways [10]. MiR-146a induction is stimulated by toll-like receptors (TLRs), interleukin (IL)-1β and tumour necrosis factor (TNF)- α. They primarily target IL receptor associated kinase 1 (IRAK1) and TNF receptor associated factor 6 (TRAF6) to modulate and prevent overstimulation of inflammatory responses in the TLR/NF-κB pathways [11]. The miR-146a G/C SNP (due to a C:U miss-pairing taking place instead of a normal G:U pairing), contributes towards the pathogenesis of several inflammatory diseases, including autoimmune disorders [12], sepsis [13], cardiovascular disease [14] and diabetes [15]. This SNP is situated within the crucial stem region of pre-miRNA-146a and affects the expression of mature miR-146a [16]. The miR-146a rs2910164 is also associated with psoriasis [17] and PsA [18], however limited data are available.

In South Africa, psoriasis and PsA is extremely rare among the indigenous African population while such cases are more common in both the Indian and Caucasian population [19]. Taking this into consideration, the present study investigated whether rs2910164 contributes towards psoriasis and PsA development in South African Indian and Caucasian patients. This was done by comparing the rs2910164 genotype and allele frequency distribution between the Caucasian and Indian PsA patients and healthy controls for any similarities or deviations.

## Methods

### Patient recruitment and sample collection

Blood samples were taken from South African Indian (*n* = 84) and Caucasian (*n* = 32) PsA patients (total *n* = 116) and healthy control subjects (Indian: *n* = 62 and Caucasian: *n* = 38; total *n* = 100) that were enrolled in the study after informed consent following ethical approval from the Pharma-Ethics Research Ethics Committee (ethics reference number: 13095660). The rheumatoid factor-immunoglobulin M (RF-IgM), C-reactive protein (CRP), plasma glucose, blood glycated haemoglobin (HbA1c), total cholesterol, low density lipoprotein (LDL) cholesterol, high density lipoprotein (HDL) cholesterol and Vitamin D25 levels were assessed at Lancet Laboratories (Durban, South Africa), a fully accredited South African National Laboratory. The Health Assessment Questionnaire (HAQ), an assessment tool used for measuring the overall functional health status of patients with PsA, was used to generate HAQ scores. Briefly, the HAQ Disability Index (HAQ-DI) was used to assess the level of functional ability in patients. The HAQ visual analogue (VAS) pain scale was used to assess the absence or presence of PsA related pain and its severity in patients. The HAQ VAS patient global health scale was used to assess the overall quality of life for patients where 0 = good health and 10 = poor health. HAQ score values < 0.5 and > 0.5 indicated patients had minimal functional impairments and moderate to severe functional impairments from the PsA, respectively. The physical measurements of height, weight, and abdominal and waist circumference, and patient history (age, sex, race, disease duration, radiology, HAQ scores and medications) were conducted by the consultant rheumatologist. The inclusion criteria for this study, irrespective of age and gender, were: (a) patients must be over the age of 18 years and have PsA; (b) patients must have a confirmed diagnosis of PsA and must have fulfilled the Classification Criteria for PsA (CASPAR) [20] criteria (patients with all other forms of inflammatory arthritis were excluded from this study); (c) Patients with all other forms of connective tissue disorders were excluded from this study.

### DNA extraction

Genomic DNA was extracted from whole blood taken from PsA patients and controls using the FlexiGene® DNA isolation kit (Qiagen). Briefly, 750 μl cell lysis buffer was added to 300 μl whole blood to pellet out the mitochondria and cell nuclei, followed by the removal of contaminants such as proteins in the pellet by adding 150 μl denaturation buffer, which contained a chaotropic salt and protease enzyme, and incubating for 5 min at 65 °C. To this solution, 150 μl 100% isopropanol was added to precipitate out the DNA and was recovered by centrifugation. The DNA was washed in 150 μl 100% ethanol, dried at room temperature, resuspended in 15 μl hydration buffer (10 mM Tris.Cl, pH 8.5), incubated for 1 h at 65 °C and stored at -20 °C until further use. The Nanodrop 2000 spectrophotometer (Thermo Scientific) was used to determine the purity and concentration of the DNA. All DNA samples were standardised to a concentration of 10 ng/μl.

### Genotyping

The miR-146a G/C rs2910164 was genotyped using polymerase chain reaction-restriction fragment length polymorphism (PCR-RFLP). The GoTaq® G2 Flexi DNA Polymerase PCR kit (Promega) and the CFX96 Touch™ Real-Time PCR Detection System (Bio-Rad) was used for this analysis. The 147 bp gene amplicon was amplified using 1× Green GoTaq Flexi buffer, 2.5 mM MgCl_2_, 200 μM of each dNTP, 0.2 Units GoTaq Flexi DNA polymerase, 20 pmol of each forward (F) and reverse (R) primer sequences, and 30 ng genomic DNA template. A no-template control was run with the positive samples to assess the overall specificity of the reaction. The forward and reverse primer sequences used were 5′-CATGGGTTGTGTCAGTGTCAGAGCT-3′, and 5′-TGCCTTCTGTCTCCAGTCTTCCAA-3′, respectively. PCR conditions were: 94 °C for 10 min (initial denaturation), followed by 30 cycles at 94 °C for 30 s (denaturation), 65 °C for 30 s (annealing) and 72 °C for 30 s (extension), and 72 °C for 7 min (final extension). The 147 bp PCR products were electrophoresed on 1.8% agarose gel containing 2 μl GelRed and visualised using the ChemiDoc™ XRS+ Imaging System (Bio-Rad). The *Sac* I restriction enzyme (New England BioLabs) was used to digest the PCR products at 37 °C for 16 h, electrophoresed on 3% agarose gel containing 2 μl GelRed and visualised as mentioned above. Presence of the homozygous wild-type G-allele (GG genotype) resulted in no cleavage of the 147 bp PCR product. The homozygous variant C-allele (CC genotype) yielded two fragments of 122 and 25 bp. The heterozygous GC genotype yielded three bands of 147, 122 and 25 bp. A DNA ladder was used to accurately determine the different genotypes.

### Statistical analysis

The post-hoc power analysis was used to calculate the overall statistical power of the present study [21, 22]. All statistical analysis was performed using the IBM SPSS statistical software (version 24) and GraphPad Prism software (version 5.0) packages. The Student’s unpaired *t*-test was used to compare the characteristics of PsA patients and the control groups (Table 1). The Chi-squared (χ^2^) test and Fisher’s exact test were used to analyse the genotype and allele frequencies, respectively (Table 2 and Table 3). The χ^2^ test was also used to assess whether the genotype frequencies complied with the Hardy-Weinberg equilibrium. The Fisher’s exact test data are represented as the relative risk ratio (RR) and odds ratio (OR) at 95% confidence intervals (CI). Data were expressed as mean ± standard error (Table 1). A *p* value less than 0.05 was considered statistically significant.

**Table 1:** Clinical and demographical characteristics of PsA patients and controls

	PsA patients (*n* = 117)	Controls (*n* = 100)	*p* value
Age (years)	50.34 ± 1.14	46.23 ± 1.56	0.0309^*^
Sex			
Male, n (%)	63 (54)	35 (35)	
Female, n (%)	54 (46)	65 (65)	
Race			
Indian, n (%)	84 (72)	62 (62)	
White, n (%)	32 (27)	38 (38)	
Mixed Race, n (%)^b^	1 (1)	0 (0)	
BMI (kg/m^2^)	28.86 ± 0.50	27.85 ± 0.42	0.1307
Smoker			
Yes, n (%)	25 (21)		
No, n (%)	92 (79)		
Disease duration (years)	6.43 ± 0.67		
Radiology			
Yes, n (%)	84 (72)		
No, n (%)	33 (28)		
HAQ score	0.62 ± 0.07		
RF-IgM			
Positive, n (%)	5 (4)		
Negative, n (%)	112 (96)		
Drugs			
MTX, n (%)	111 (95)		
SSZ, n (%)	33 (28)		
LFM, n (%)	21 (18)		
Biologics, n (%)^a^	9 (8)		
CRP (mg/L)			
Inclusion	18.95 ± 2.81		0.0011^*^
@ 6 month	9.68 ± 1.32		
Plasma glucose (mmol/l)	6.17 ± 0.21		
Total cholesterol (mmol/l)	5.11 ± 0.10		
LDL cholesterol (mmol/l)	3.26 ± 0.12		
HDL cholesterol (mmol/l)	1.14 ± 0.03		
HbA1c (%)	5.85 ± 0.13		
Vitamin D25 (ng/ml)	20.31 ± 1.34		

Laboratory parameters are represented as mean ± standard error. Comparisons for age, BMI and CRP levels were performed using the unpaired Student’s *t*-test

*PsA* psoriatic arthritis, *BMI* body mass index, *HAQ* health assessment questionnaire, *RF-IgM* rheumatoid factor-immunoglobulin M, *MTX* methotrexate, *SSZ* sulfasalazine, *LFM* leflunomide, *CRP* C-reactive protein, *LDL* low density lipoprotein, *HDL* high density lipoprotein, *HbA1c*: blood glycated haemoglobin

^*^A *p* < 0.05 was considered as being significant

^a^Biologics: etanercept, adalimumab and infliximab. ^b^Mixed Race: Caucasian and Indian descent

**Table 2:** Genotype and allele frequencies for all Indians and Caucasians (PsA patients + controls combined)

Frequency, n (%)	Indians (*n* = 146)^d^	Caucasians (*n* = 70)^e^	*p* value	RR (95% CI)	OR (95% CI)
Genotype, n (%)					
GG	68 (46.57)	27 (38.57)	0.4536^a^		
GC	68 (46.57)	39 (55.71)			
CC	10 (6.85)	4 (5.71)			
GC + CC	78 (53.42)	43 (61.43)	0.3064^b^	1.11 (0.92–1.33)	1.39 (0.78–2.48)
Allele, n (%)					
G	204 (69.86)	93 (66.43)	0.5063^c^	1.05 (0.91–1.22)	1.17 (0.76–1.08)
C	88 (30.14)	47 (33.57)			

*CI* confidence interval, *DF* degrees of freedom, *RR* relative risk, *OR* odds ratio, *PsA* psoriatic arthritis, χ^2^ Chi-squared test

A *p* < 0.05 was considered as being significant

^a^Chi squared p value (Indians genotypes vs. Caucasians genotypes); χ^2^ = 1.581, 2 DF. ^b^Fisher’s exact test *p* value (GG vs. GC + CC genotypes). ^c^Fisher’s exact test *p* value (Indians G and C alleles vs. Caucasians G and C alleles). ^d^Indians (84 Indian PsA patients + 62 Indian controls). ^e^Caucasians (32 Caucasian PsA patients + 38 Caucasian controls)

**Table 3:** Genotype and allele frequencies for PsA patients and controls before and after stratification for race (Indians and Caucasians)

Frequency, n (%)	Controls	PsA patients	*p* value	RR (95% CI)	OR (95% CI)
Unstratified: Indians + Caucasians (PsA patients: *n* = 116 and controls: *n* = 100)					
Genotype, n (%)					
GG	52 (52)	43 (37.07)	0.0595^a1^		
GC	44 (44)	63 (54.31)			
CC	4 (4)	10 (8.62)			
GC + CC	48 (48)	73 (62.93)	0.0290^b^	1.38 (1.04–1.84)	1.84 (1.07–3.17)
Allele, n (%)					
G	148 (74)	149 (64.22)	0.0295^c^	1.29 (1.02–1.65)	1.59 (1.05–2.40)
C	52 (26)	83 (35.78)			
Stratified: Indians (PsA patients: *n* = 84 and controls: *n* = 62)					
Genotype, n (%)					
GG	37 (59.68)	31 (36.90)	0.0241^a2^		
GC	22 (35.48)	46 (54.76)			
CC	3 (4.84)	7 (8.33)			
GC + CC	25 (40.32)	53 (63.10)	0.0075^b^	1.70 (1.15–2.51)	2.53 (1.29–4.96)
Allele, n (%)					
G	96 (77.41)	108 (64.29)	0.0200^c^	1.48 (1.05–2.08)	1.91 (1.13–3.22)
C	28 (22.58)	60 (35.71)			
Stratified: Caucasians (PsA patients: *n* = 32 and controls: *n* = 38)					
Genotype, n (%)					
GG	15 (39.47)	12 (37.50)	0.4793^a3^		
GC	22 (57.89)	17 (53.13)			
CC	1 (2.63)	3 (9.38)			
GC + CC	23 (60.52)	20 (62.5)	0.9383^b^	1.04 (0.67–1.61)	1.09 (0.41–2.86)
Allele, n (%)					
G	52 (68.42)	41 (64.06)	0.5955^c^	1.10 (0.78–1.53)	1.22 (0.60–2.46)
C	24 (31.58)	23 (35.04)			

*CI* confidence interval, *DF* degrees of freedom, *RR* relative risk, *OR* odds ratio, *PsA* psoriatic arthritis, χ^2^ Chi-squared test

^a1, a2, a3^Chi squared *p* value (controls genotypes vs. PsA patients genotypes) (^a1^ χ^2^ = 5.644, 2 DF; ^a2^ χ^2^ = 7.454, 2 DF; ^a3^ χ^2^ = 1.471, 2 DF). ^b^Fisher’s exact test *p* value (GG vs. GC + CC genotypes). ^c^Fisher’s exact test *p* value (controls G and C alleles vs. PsA patients G and C alleles). A *p* < 0.05 was considered as being significant

## Results

The overall statistical power of this study (based on the post-hoc power analysis) was low (19%), however the sample size was adequate to compare the miR-146a G/C rs2910164 genotype and allele frequency distribution between the Caucasian and Indian PsA patients and healthy controls for any similarities or deviations.

The demographics and clinical characteristics of all study subjects are shown in Table 1. There was a significant difference between patients and controls regarding age (*p* = 0.0309) but not BMI (*p* = 0.1307). Patients displayed moderate to severe functional impairments from the PsA (HAQ score = 0.62 ± 0.07), and over 96% tested negative for the RF-IgM. Majority of patients (95%) were on methotrexate (MTX), and a significant reduction in CRP levels at inclusion (18.95 ± 2.81 mg/L) and after 6 months (9.68 ± 1.32 mg/L) was observed (*p* = 0.0011). Blood glucose (6.17 ± 0.21 mmol/l) and HbA1c (5.85 ± 0.13%) levels were characteristics of pre-diabetes and impaired fasting glucose. Additionally, patients had lower HDL (1.14 ± 0.03 mmol/l) and higher LDL (3.26 ± 0.12 mmol/l) cholesterol levels.

The genotype and allele frequency distribution for all Caucasians and Indians (PsA patients + healthy controls combined) are shown in Table 2. Caucasians and Indians had similar genotype (*p* = 0.4536) and allele (*p* = 0.5063) frequency distribution. Caucasians had a slightly higher frequency of the GC + CC genotypes compared to Indians, but this was not statistically significant (61.43% vs. 53.42% respectively, *p* = 0.3064, OR = 1.39 95% CI 0.78–2.48).

Table 3 highlights the genotype and allele frequency distribution for PsA patients and healthy controls before and after stratification for race. ***Unstratified data (Caucasians + Indians)*:** There was almost a significant genotype distribution between PsA patients and healthy controls (GG, GC, CC: 37.07%, 54.31%, and 8.62% versus 52%, 44%, and 4%; *p* = 0.0595). PsA patients had a significantly higher frequency of the GC + CC genotypes (62.93% vs. 48%, *p* = 0.0290, OR = 1.84 95% CI 1.07–3.17) and variant C-allele (35.78% vs. 26%, *p* = 0.0295, OR = 1.59 95% CI 1.05–2.40) compared to healthy controls. ***Stratified data (Indians)*:** There was a significant genotype distribution between Indian PsA patients and healthy Indian controls (GG, GC, CC: 36.90%, 54.76%, and 8.33% versus 59.68%, 35.48%, and 4.84%; *p* = 0.0241). Indian PsA patients had a significantly higher frequency of the GC + CC genotypes (63.10% vs. 40.32%, *p* = 0.0075, OR = 2.53 95% CI 1.29–4.96) and variant C-allele (35.71% vs. 22.58%, *p* = 0.0200, OR = 1.91 95% CI 1.13–3.22) compared to healthy Indian controls. ***Stratified data (Caucasians)*:** No significance was noted; Caucasian and Indian PsA patients had similar genotype and allele frequency distribution.

## Discussion

In this study, we evaluated the frequency of the miR-146a G/C rs2910164 in South African Indian and Caucasian patients with PsA compared to healthy control subjects. We observed a significantly higher prevalence of the variant C-allele in Indian PsA patients compared to healthy Indian controls (35.71% vs. 22.58% respectively, *p* = 0.0200, OR = 1.91 95% CI 1.13–3.22). Conversely, the variant C-allele frequency between Caucasian PsA patients and healthy controls were similar. Data suggests that Indian PsA patients with the heterozygous GC and homozygous variant CC genotypes (GC + CC) are more predisposed to developing PsA compared to patients with the homozygous wild-type GG genotype.

Patients with uncontrolled psoriasis and PsA have exacerbated CRP levels [23, 24]. CRP is a biomarker of inflammation and disease severity. Psoriasis, PsA and rheumatoid arthritis are characterised by the secretion of several pro-inflammatory cytokines such as IL-2, IL-6, IL-8, IFN-γ and TNF-α. These cytokines are responsible for producing high levels of CRP and contributing to the pathophysiology of the disease. TNF-α induced secretion of IL-6 activates the production of CRP by stimulating the transcription of the CRP gene via activation of the IRAK1 and signal transducer and activator of transcription 3 (STAT3) inflammatory pathways; thereby, causing the high CRP levels observed in psoriasis, PsA and rheumatoid arthritis [25–27]. The PsA patients recruited in this study had high CRP levels (Table 1).

When miR-146a is highly expressed, it inhibits both IRAK1 and TRAF6 resulting in concomitant reductions in pro-inflammatory cytokines (IL-2, IL-6, IL-8, IFN-γ and TNF-α) expression and CRP levels [14]. The rs2910164 variant C-allele dampens the overall functionality of miR-146a, leading to an upregulation in IRAK1 and TRAF6 expression, resulting in very high cytokine production [13].

In an Egyptian cohort, patients with psoriasis had abnormally higher miR-146a expression, and MTX treatment significantly reduced miR-146a expression [28]. Over 96% of PsA patients in our study received MTX that resulted in a significant decrease in CRP levels (CRP monitored at inclusion versus after 6 months) after treatment (*p* = 0.0011) (Table 1).

In 2014, Zhang et al. reported that Chinese patients homozygous for the wild-type GG genotype and heterozygous for the GC genotypes (GG + GC) compared to patients homozygous for the variant CC genotype had a greater risk of developing psoriasis and PsA [17]. The frequency of the wild-type G-allele was more common in psoriasis patients compared to healthy controls (48.2% versus 42.4%; *p* = 0.007). The frequency of the GG (21.7%) and GC (53.0%) genotypes were predominant among psoriasis patients versus controls (GG: 16.7% and GC: 51.5%) whereas the CC genotype was more common in the healthy controls versus psoriasis patients (31.8% versus 25.3%). The difference in the distribution of the rs2910164 genotypes between psoriasis patients and controls were statistically significant (*p* = 0.021). The combined frequency of the GG + GC genotypes were significantly higher among PSA patients compared to the controls (74.7% versus 68.2%; *p* = 0.018), and was associated with an increase in PsA susceptibility (adjusted OR = 1.38 95% CI 1.06–1.80) [17]. Chatzikyriakidou et al. (2010) found the frequency of the GC genotype to be higher in Greek PsA patients compared to healthy controls (41.4% versus 27.3%). The GG and CC genotypes were higher in the controls compared to PsA patients (GG: 59.1% versus 48.3% and CC: 13.6% versus 10.3%). However, no significant difference was observed in the distribution of the rs2910164 genotypes between PsA patients and controls (*p* = 0.394) [18].

In our study, the frequency of the GC + CC genotypes and variant C-allele were significantly higher in all PsA patients versus all healthy controls, with no significant changes in genotype distribution between patients and controls. No association between rs2910164 and PsA were noted in the Caucasian population. There was a significant difference in the distribution of the rs2910164 genotypes between Indian PsA patients and controls. Indian PsA patients had a significantly higher frequency of the GC + CC genotypes and variant C-allele versus healthy controls (Table 3).

Psoriasis and PsA patients have a higher prevalence of MetS [29]. In 2012, Langhan et al. reported that patients with mild, moderate and severe psoriasis and PsA had a 22%, 56% and 98% chance of developing MetS, respectively [30]. PsA patients recruited in our study had a mean HAQ score indicative of moderate to severe functional impairments from the disease, and displayed increased fasting plasma glucose and HbA1c levels indicative of early stages of pre-diabetes and impaired fasting glucose (Table 1). Smoking, diet and obesity are some of the environmental risk factors associated with psoriasis and PsA [1]. Cigarette smoke affects the central nervous system and immune system, and causes pro-inflammatory and anti-inflammatory cytokines to be aberrantly expressed [1]. Nicotine binds to the nicotinic acetylcholine receptors (nAChR) found in several cell types, including immune B-cells, T-cells, thymocytes and leukaemic cell lines. This can lead to defective immune and nervous system signalling processes, and can negatively regulate keratinocyte function [1, 31]. Apart from psoriasis and PsA, smoking can also play an insidious role in triggering MetS [32]. In our study, PsA patients had a mean BMI indicating overweight and obesity, and over 21% were active smokers (Table 1).

## Conclusion

This study associated the rs2910164 with increased PsA susceptibility in the South African Indian population. A major limitation of the study is the small sample size in the case-control cohorts, with a low overall statistical power (post-hoc power analysis = 19%). The influence of rs2910164 on miR-146a expression and its role in the pathogenesis of PsA necessitates investigation in a bigger cohort.

## Abbreviations

BMI: Body mass index
C: Cytosine
CI: Confidence interval
CRP: C reactive protein
DF: Degrees of freedom
G: Guanine
HAQ: Health assessment questionnaire
HbA1c: Glycated haemoglobin
HDL: High density lipoprotein
HWE: Hardy-Weinberg equilibrium
IL: Interleukin
IRAK1: Interleukin receptor associated kinase 1
LDL: Low density lipoprotein
LFM: Leflunomide
MetS: Metabolic syndrome
MiR: MicroRNA
MTX: Methotrexate
OR: Odds ratio
PsA: Psoriatic arthritis
RF-IgM: Rheumatoid factor-immunoglobulin M
RR: Risk ratio
SNP: Single nucleotide polymorphism
SSZ: Sulfasalazine
TNF-α: Tumour necrosis factor alpha
TRAF6: Tumour necrosis factor-α receptor associated factor 6
UTR: Untranslated region
χ^2^: Chi-squared

## References

[1] Zeng J, Luo S, Huang Y, Lu Q (2017). Critical role of environmental factors in the pathogenesis of psoriasis. J Dermatol..

[2] Gelfand JM, Neimann AL, Shin DB, Wang X, Margolis DJ, Troxel AB (2006). Risk of myocardial infarction in patients with psoriasis. JAMA.

[3] Coates LC, FitzGerald O, Helliwell PS, Paul C (2016). Psoriasis, psoriatic arthritis, and rheumatoid arthritis: is all inflammation the same?. Semin Arthritis Rheum.

[4] Gelfand JM, Gladman DD, Mease PJ, Smith N, Margolis DJ, Nijsten T, Stern RS, Feldman SR, Rolstad T (2005). Epidemiology of psoriatic arthritis in the population of the United States. J Am Acad Dermatol.

[5] Alamanos Y, Voulgari PV, Drosos AA (2008). Incidence and prevalence of psoriatic arthritis: a systematic review. J Rheumatol.

[6] Taylor WJ (2002). Epidemiology of psoriatic arthritis. Curr Opin Rheumatol.

[7] Cannell Ian G, Kong Yi W, Bushell M (2008). How do microRNAs regulate gene expression?. Biochem Soc Trans.

[8] Bartel DP (2004). MicroRNAs. Cell.

[9] Liu X, Han Z, Yang C (2017). Associations of microRNA single nucleotide polymorphisms and disease risk and pathophysiology. Clin Genet.

[10] Omrane I, Kourda N, Stambouli N, Privat M, Medimegh I, Arfaoui A, Uhrhammer N, Bougatef K, Baroudi O, Bouzaienne H (2014). MicroRNAs 146a and 147b biomarkers for colorectal Tumor's localization. Biomed Res Int.

[11] Saba R, Sorensen DL, Booth SA (2014). MicroRNA-146a: a dominant, negative regulator of the innate immune response. Front Immunol.

[12] De Felice B, Manfellotto F, Palumbo A, Troisi J, Zullo F, Di Carlo C, Sardo ADS, De Stefano N, Ferbo U, Guida M (2015). Genome–wide microRNA expression profiling in placentas from pregnant women exposed to BPA. BMC Med Genet.

[13] Shao Y, Li J, Cai Y, Xie Y, Ma G, Li Y, Chen Y, Liu G, Zhao B, Cui L (2014). The functional polymorphisms of miR-146a are associated with susceptibility to severe sepsis in the Chinese population. Mediat Inflamm.

[14] Ramkaran P, Khan S, Phulukdaree A, Moodley D, Chuturgoon AA (2014). miR-146a polymorphism influences levels of miR-146a, IRAK-1, and TRAF-6 in young patients with coronary artery disease. Cell Biochem Biophys.

[15] Alipoor B, Meshkani R, Ghaedi H, Sharifi Z, Panahi G, Golmohammadi T (2016). Association of miR-146a rs2910164 and miR-149 rs2292832 variants with susceptibility to type 2 diabetes. Clin Lab.

[16] Jazdzewski K, Murray EL, Franssila K, Jarzab B, Schoenberg DR, de la Chapelle A (2008). Common SNP in pre-miR-146a decreases mature miR expression and predisposes to papillary thyroid carcinoma. Proc Natl Acad Sci.

[17] Zhang W, Yi X, Guo S, Shi Q, Wei C, Li X, Gao L, Wang G, Gao T, Wang L (2014). A single-nucleotide polymorphism of miR-146a and psoriasis: an association and functional study. J Cell Mol Med.

[18] Chatzikyriakidou A, Voulgari P, Georgiou I, Drosos A (2010). The role of microRNA-146a (miR-146a) and its target IL-1R-associated kinase (IRAK1) in psoriatic arthritis susceptibility. Scand J Immunol.

[19] Maharaj AB, Tak PP (2015). Spondyloarthritis in African blacks. J Rheumatol.

[20] Taylor W, Gladman D, Helliwell P, Marchesoni A, Mease P, Mielants H (2006). Classification criteria for psoriatic arthritis: development of new criteria from a large international study. Arthritis & Rheumatology.

[21] Post-hoc Power Calculator: Evaluate statistical power of an existing study. http://clincalc.com/stats/power.aspx. Accessed 20 Nov 2017.

[22] Levine M, Ensom MH (2001). Post hoc power analysis: an idea whose time has passed?. Pharmacotherapy.

[23] Beygi S, Lajevardi V, Abedini R (2014). C-reactive protein in psoriasis: a review of the literature. J Eur Acad Dermatol Venereol.

[24] Sudhesan A, Rajappa M, Chandrashekar L, Ananthanarayanan PH, Thappa DM, Satheesh S, Chandrasekaran A, Devaraju P (2016). Association of C-reactive protein (rs1205) gene polymorphism with susceptibility to psoriasis in south Indian Tamils. J Clin Diagn Res.

[25] Hashemi M, Eskandari-Nasab E, Zakeri Z, Atabaki M, Bahari G, Jahantigh M, Taheri M, Ghavami S (2013). Association of pre-miRNA-146a rs2910164 and pre-miRNA-499 rs3746444 polymorphisms and susceptibility to rheumatoid arthritis. Mol Med Rep.

[26] Hassine HB, Boumiza A, Sghiri R, Baccouche K, Boussaid I, Atig A, Shakoor Z, Bouajina E, Zemni R (2017). Micro RNA-146a but not IRAK1 is associated with rheumatoid arthritis in the Tunisian population. Genet Test Mol Biomarkers.

[27] Farshchian M, Ansar A, Sobhan M, Hoseinpoor V (2016). C-reactive protein serum level in patients with psoriasis before and after treatment with narrow-band ultraviolet B. An Bras Dermatol.

[28] Ele-Refaei AM, El-Esawy FM (2015). Effect of narrow-band ultraviolet B phototherapy and methotrexate on MicroRNA (146a) levels in blood of psoriatic patients. Dermatol Res Pract.

[29] Carvalho AV, Romiti R, Souza CD, Paschoal RS, Milman LD, Meneghello LP (2016). Psoriasis comorbidities: complications and benefits of immunobiological treatment. An Bras Dermatol.

[30] Langan SM, Seminara NM, Shin DB, Troxel AB, Kimmel SE, Mehta NN, Margolis DJ, Gelfand JM (2012). Prevalence of metabolic syndrome in patients with psoriasis: a population-based study in the United Kingdom. J Investig Dermatol.

[31] Moriwaki Y, Takada K, Tsuji S, Kawashima K, Misawa H (2015). Transcriptional regulation of SLURP2, a psoriasis-associated gene, is under control of IL-22 in the skin: a special reference to the nested gene LYNX1. Int Immunopharmacol.

[32] Slagter SN, van Vliet-Ostaptchouk JV, Vonk JM, Boezen HM, Dullaart RP, Kobold AC, Feskens EJ, van Beek AP, van der Klauw MM, Wolffenbuttel BH (2013). Associations between smoking, components of metabolic syndrome and lipoprotein particle size. BMC Med.

